# Lean-to-Rich Healthy Aging: A New Hypothesis on Okinawa’s Postwar Nutrition Transition and Longevity Trends

**DOI:** 10.3390/nu18203415

**Published:** 2026-10-18

**Authors:** Yuzuru Ohshiro

**Affiliations:** Department of Diabetology and Endocrinology, Omoromachi Medical Center, Tokushukai Medical Group, Naha 900-0011, Okinawa, Japan; ooshiro@aol.com

**Keywords:** Okinawa, longevity, healthy aging, nutrition transition, life-course nutrition, nutritional trajectory, body mass index, cohort effect

## Abstract

This review proposes a new life-course nutritional hypothesis generated from Okinawa’s unique postwar history and changing longevity advantage relative to mainland Japan. After World War II, Okinawa underwent rapid socioeconomic and dietary change and experienced a nutrition transition earlier than mainland Japan. During subsequent decades, Okinawa developed a marked relative longevity advantage, which later diminished as longevity in mainland Japan continued to improve. We propose that this pattern may partly reflect differences in the timing and sequence of life-course nutritional environments. Older Okinawan cohorts experienced an initially relatively Lean nutritional environment followed by postwar nutritional enrichment, approximating a Lean-to-Rich trajectory. Comparable trajectories may have emerged later in mainland Japan, potentially contributing to national catch-up, while progressively younger Okinawan cohorts experienced the post-transition environment from earlier life stages, increasingly approximating a Rich-to-Rich trajectory. Mortality reanalysis confirmed an older-age advantage and a progressive generational gradient. We therefore propose the Lean-to-Rich Healthy Aging Hypothesis, distinguishing three conceptual trajectories: persistent relative nutritional restraint (Lean-to-Lean), transition from an initially Lean to a subsequently Rich nutritional environment (Lean-to-Rich), and prolonged nutritional abundance (Rich-to-Rich). We hypothesize that Lean-to-Rich may be more favorable for healthy longevity than either persistent Lean-to-Lean or prolonged Rich-to-Rich. The hypothesis does not specify the optimal timing, magnitude, duration, or composition of the transition, and available ecological evidence cannot distinguish a sequence effect from cumulative Rich exposure. Rather than establishing causality, this framework generates testable predictions concerning how the sequence and duration of nutritional environments across the life course may influence healthy longevity.

## 1. Introduction

Okinawa has long been recognized internationally as a region of exceptional longevity. Older Okinawan generations, particularly those born before World War II, have exhibited low mortality and a high prevalence of survival to advanced age [1,2].

The traditional Okinawan diet, historically characterized by relatively low energy intake and a high proportion of plant-derived foods, has frequently been proposed as one contributor to this longevity phenotype [2]. Long-term analyses of older Okinawan cohorts have documented relatively low caloric intake, low body mass index, low mortality from age-related diseases, and survival patterns consistent with extended longevity [2].

Other potential contributors include genetic and familial factors, physical activity, and broader lifestyle and psychosocial characteristics [3,4,5].

However, the historical pattern of Okinawan longevity is more complex than a simple narrative of persistent exceptional longevity. Evidence regarding the prewar period is limited and methodologically heterogeneous. Poulain and Herm concluded that available prewar life tables did not demonstrate a clear and consistent longevity advantage for Okinawa relative to mainland Japan [1]. The historical prefectural life tables compiled by Mizushima (1921–1956) show period- and sex-specific variation, with some estimates favorable to Okinawa, particularly among women [6]. These observations do not necessarily conflict: favorable estimates in particular periods or sexes do not by themselves establish a consistent prewar Okinawan longevity advantage, and differences in data quality, registration completeness, and life-table methodology complicate direct comparisons [1,6]. The present hypothesis does not depend on whether Okinawa had a longevity advantage before World War II; it rests on the postwar pattern described below.

In this article, longevity is the principal historical outcome because comparable long-term population data on frailty, functional capacity, and disability-free survival are not available for the relevant decades. “Healthy aging” denotes the broader outcome that the hypothesis ultimately seeks to explain and must be tested using both survival and functional-health endpoints.

A methodologically consistent picture is available from the official Prefectural Life Tables of the Ministry of Health, Labour and Welfare from 1975 onward [7] (Figure 1).

Among men, the difference in life expectancy at birth between Okinawa and the national average increased from +0.36 years in 1975 to +1.39 years in 1985, but subsequently declined, became negative around 2000, and reached −0.76 years in 2020. Among women, the corresponding advantage increased from +1.95 years in 1975 to +2.95 years in 1985 and then progressively narrowed to +0.28 years by 2020 [7].

Okinawan life expectancy nevertheless continued to increase; the relative advantage narrowed because improvements elsewhere in Japan increasingly matched or exceeded those in Okinawa [7]. The postwar Okinawan experience is therefore characterized not by a decline in longevity, but by the emergence, amplification, and subsequent convergence of a relative longevity advantage.

## 2. Okinawa’s Earlier Postwar Nutrition Transition

Following World War II, Okinawa remained under U.S. administration until 1972 and experienced rapid socioeconomic and dietary change: food availability expanded, imported foods became increasingly accessible, and consumption of meat, animal products, and oils increased [8].

Importantly, this transformation occurred earlier in Okinawa than in mainland Japan. Historical dietary data summarized by Todoriki and colleagues indicate that the increase in the proportion of dietary energy derived from fat preceded the corresponding national transition by approximately a decade [8] (Figure 2). We use this shift as a population-level marker of a broader nutrition transition, not as the causal exposure itself or as a claim that mainland Japan remained absolutely Lean.

This time lag is central to the present hypothesis. People of the same birth cohort in Okinawa and mainland Japan could experience different nutritional sequences: an Okinawan born before the transition could spend a substantial period in a relatively Lean environment before entering a Richer one, whereas a mainland Japanese person of the same birth cohort could remain in the earlier environment for longer. Conversely, later-born Okinawans would encounter the post-transition environment from progressively earlier stages of life, while comparable mainland cohorts could retain a longer preceding Lean period.

Taken together, Figure 1 and Figure 2 show two parallel temporal patterns. Okinawa underwent nutritional enrichment approximately a decade earlier than mainland Japan and subsequently developed a marked relative longevity advantage, which narrowed as mainland Japan underwent its own transition and as younger Okinawan cohorts encountered the post-transition environment from earlier stages of life [7,8]. These parallel patterns raise the possibility that the relevant exposure may not be simply a Lean or Rich nutritional state, but the sequence in which these environments are experienced. We therefore propose the Lean-to-Rich Healthy Aging Hypothesis (Section 3).

Accordingly, this review examines two principal propositions: (1) whether Lean-to-Rich may be more favorable for healthy longevity than persistent Lean-to-Lean, and (2) whether Lean-to-Rich may be more favorable than prolonged Rich-to-Rich. We first consider the Okinawan historical evidence underlying each proposition and then examine independent epidemiological evidence relevant to their plausibility. We also consider competing explanations, including cumulative duration of Rich exposure and developmental mismatch under the developmental origins of health and disease (DOHaD) framework, and identify the evidence required to distinguish among these possibilities.

### Data and Methods for the Mortality Reanalyses

Two descriptive analyses were performed using publicly available all-cause mortality data from the Japanese Mortality Database (JMD; version 005_002) for Okinawa Prefecture and Japan overall [9]. First, to quantify which ages generated the historical Okinawa–Japan life-expectancy difference, the difference was decomposed for 1975–1979, 1980–1984, and 1985–1989 using the sex-specific JMD 5-year life tables. Following established demographic decomposition methods [10,11,12], a symmetric stepwise-replacement procedure was applied to age-specific mortality schedules, averaging forward and reverse replacement orders to reduce order dependence; contributions were grouped as ages 0–4, 5–19, 20–39, 40–59, 60–74, 75–84, and ≥85 years. Second, to distinguish attained age, calendar period, and birth cohort more explicitly, sex-specific death rates tabulated by 5-year calendar period and 5-year age group were matched between Okinawa and Japan, and an Okinawa-to-Japan mortality-rate ratio was calculated for each cell. Approximate birth cohort was reconstructed as the calendar-period midpoint minus the age-group midpoint. Analyses focused on ages 50–89 years, consistent with the age range emphasized by Poulain and Herm; for each synthetic 5-year birth cohort, available ratios were averaged separately for women and men, and cohorts with at least four observations (approximately 1905–1955 births) were displayed. Both are descriptive population-level analyses, not longitudinal follow-up of individuals or causal age-period-cohort models. Because Figure 1 uses official single-year prefectural life tables while the decomposition uses JMD 5-year period life tables, small numerical differences between them reflect different time aggregation rather than inconsistency.

## 3. The Lean-to-Rich Healthy Aging Hypothesis

We propose three conceptual life-course nutritional trajectories (Figure 3).

In this framework, “Lean” and “Rich” are multidimensional relative nutritional environments rather than BMI categories or fixed caloric thresholds. Lean denotes comparatively constrained energy availability and lower cumulative exposure to energy excess, typically accompanied at the population level by lower body mass and metabolic burden. Rich denotes greater availability of energy and nutrients after socioeconomic and food-system transition and therefore spans nutritional adequacy through nutritional excess; adequacy and excess are not treated as biologically equivalent. Future studies should operationalize these dimensions separately using energy intake and availability, dietary composition and quality, protein and micronutrient adequacy, adiposity/body composition, and metabolic status.

### 3.1. Lean-to-Lean

Lean-to-Lean represents persistent relative nutritional restraint throughout much of the life course. Such an environment may limit cumulative exposure to excess energy, adiposity, insulin resistance, and other forms of metabolic burden [13,14].

However, persistent nutritional restraint could eventually become disadvantageous if nutritional and physiological reserves become insufficient as individuals age or face illness [15,16].

### 3.2. Rich-to-Rich

Rich-to-Rich represents nutritional abundance beginning relatively early and continuing for a prolonged period.

Such an environment may provide abundant energy and nutrients but also increases the possibility of prolonged exposure to excess energy intake, adiposity, metabolic syndrome, diabetes, hypertension, dyslipidemia, and other cardiometabolic risk factors [14,17].

### 3.3. Lean-to-Rich

Lean-to-Rich represents an initially relatively Lean nutritional environment followed by transition into a Richer nutritional environment.

We hypothesize that such a sequence may potentially combine advantages of the other two states: relatively low cumulative metabolic burden during the Lean phase, followed by greater nutritional adequacy and physiological reserve after transition to Rich.

The hypothesis therefore comprises two propositions, Lean-to-Rich > Lean-to-Lean and Lean-to-Rich > Rich-to-Rich, both of which are hypotheses rather than established relationships.

The framework concerns nutritional trajectory, and is not a prescription for deliberate dietary restriction followed by nutritional abundance or weight gain. The historical data do not identify a safe or optimal transition age, rate, magnitude, duration, or nutrient composition, and no population recommendation to restrict intake during early life or midlife can be derived from the hypothesis.

## 4. Why Might Lean-to-Rich Be More Favorable than Lean-to-Lean?

### 4.1. The Okinawa–Mainland Historical Contrast

The historical contrast between Okinawa and mainland Japan provides a hypothesis-generating observation. Because Okinawa underwent its postwar nutrition transition earlier, older Okinawan cohorts who had spent substantial portions of their lives in a relatively Lean environment subsequently experienced nutritional enrichment, approximating a Lean-to-Rich trajectory, whereas comparable mainland cohorts remained in the earlier, relatively Lean environment for longer [8].

The comparison is explicitly relative: mainland Japan was itself undergoing rapid postwar nutritional improvement, and the framework does not classify it as absolutely Lean during the 1970s–1980s. The hypothesis relies on the documented regional time lag in transition, not on a binary Lean-versus-Rich classification of whole populations.

Thus, during the period when Okinawa’s relative longevity advantage emerged, increased, and reached its maximum in 1985 in both sexes [7], the regional contrast can be conceptualized approximately as an earlier emergence of Lean-to-Rich trajectories in Okinawa and the persistence of relatively more Lean-to-Lean trajectories in mainland Japan. The timing does not establish that earlier nutritional enrichment caused the advantage. Nevertheless, the coexistence of an earlier nutrition transition and a subsequent relative longevity advantage is consistent with the possibility that transitioning from a Lean to a Richer nutritional environment may, under some conditions, be more favorable than remaining in a relatively Lean environment for longer.

### 4.2. Age-Specific Decomposition of the Historical Longevity Advantage

The decomposition (Data and Methods for the Mortality Reanalyses Section) did not support lower infant or childhood mortality as the source of Okinawa’s advantage. At ages 0–4 years, the contribution was slightly negative in all three periods for both sexes. In contrast, the positive difference was concentrated at older ages, particularly at 60 years and above (Figure 4). Among women in 1975–1979, the total Okinawa–Japan life-expectancy difference was +2.21 years, while ages ≥60 contributed +2.36 years; mortality below age 60 partially offset the advantage. In 1985–1989, the corresponding total difference was +2.78 years, with ages ≥60 contributing +2.66 years. Among men, the total advantage was smaller (+0.58 years in 1975–1979 and +1.17 years in 1985–1989), but the same pattern was evident: favorable older-age mortality was partly offset by less favorable mortality at younger ages.

These findings strengthen the interpretation that Okinawa’s historical longevity advantage reflected survival at older ages rather than an artifact of lower infant mortality. They do not identify nutrition as the cause of that survival advantage, but they establish that the demographic phenomenon underlying the hypothesis was concentrated in the age range most relevant to longevity.

### 4.3. Evidence from Body Mass and Mortality in Older Adults

A large meta-analysis by Winter and colleagues included 32 studies and 197,940 community-dwelling adults aged 65 years and older [18]. Compared with a BMI of 23.0–23.9 kg/m^2^, mortality increased progressively at lower BMI levels, whereas overweight was not associated with increased mortality. Specifically, mortality risk was already increased at BMI 21.0–21.9 and increased further at BMI 20.0–20.9 [18].

Thus, BMI values within or near the conventionally defined normal range for younger adults were associated with increased mortality in older adults. The association was not substantially altered in several sensitivity analyses, although important concerns regarding reverse causation and smoking remain [18].

Other cohort studies and meta-analyses have similarly suggested that the BMI range associated with the lowest mortality may shift upward with age, with both low BMI and marked obesity associated with increased risk [18].

These findings do not prove that intentional weight gain improves survival, and BMI cannot be used as a direct proxy for nutritional trajectory: it does not distinguish adipose tissue from lean mass, and reverse causation from chronic disease, smoking, selective survival, and loss of muscle mass remain important considerations [14,18,19]. These studies therefore support only the broader proposition that persistent nutritional restraint or low physiological reserve may not remain optimal throughout the life course; they do not directly demonstrate a benefit of transitioning from Lean to Rich.

Nevertheless, they challenge the assumption that the nutritional or body-reserve state associated with low metabolic burden earlier in life necessarily remains optimal indefinitely.

### 4.4. Nutritional and Physiological Reserve

Aging is associated with declining physiological reserve and increased vulnerability to malnutrition, dehydration, loss of muscle mass, and illness-related nutritional deterioration. Clinical nutrition guidelines therefore emphasize early identification of malnutrition risk and adequate nutritional intake in older persons [16]. The ESPEN guideline specifically recommends routine screening for malnutrition in older people and generally cautions against unnecessary dietary restriction; weight-reducing diets are recommended only selectively in obese older persons with weight-related health problems and in combination with physical exercise [16].

Under these circumstances, inadequate energy and protein availability may increase vulnerability to frailty, functional decline, and adverse clinical outcomes [15,16]. Adequate nutritional support is therefore considered an important component of maintaining nutritional status and function in older adults [16].

Thus, a Richer nutritional environment might become beneficial when it represents nutritional adequacy and physiological reserve, rather than chronic metabolic excess.

The first component of the Lean-to-Rich hypothesis therefore does not suggest that Lean is harmful. Instead, it proposes that the balance between metabolic restraint and physiological reserve may change over time.

Together, Okinawa’s historical trajectory and the wider aging literature support the possibility that Lean-to-Rich may be more favorable than persistent Lean-to-Lean. However, this proposition remains indirect and requires individual-level testing.

## 5. Why Might Lean-to-Rich Be More Favorable than Rich-to-Rich?

### 5.1. Synthetic Birth-Cohort Pattern and Nutrition-Transition Timing

The regional timing difference in postwar nutrition transition generates a specific cohort prediction. Older Okinawans born well before the transition experienced a substantial preceding Lean period before entering the post-transition environment, whereas successively later-born Okinawans encountered that environment at younger ages and for longer durations. If life-course trajectory matters, relative Okinawan mortality should therefore become less favorable across successively later birth cohorts.

The JMD reanalysis showed a marked generational gradient. Among synthetic cohorts born approximately in 1905, 1910, 1915, 1920, 1925, 1930, 1935, 1940, 1945, 1950, and 1955, mean female Okinawa-to-Japan mortality-rate ratios at ages 50–89 were 0.678, 0.727, 0.760, 0.795, 0.854, 0.880, 0.907, 0.969, 1.019, 1.027, and 1.118, respectively. Corresponding male ratios were 0.786, 0.825, 0.864, 0.890, 0.932, 0.917, 0.927, 0.981, 1.057, 1.136, and 1.263. Thus, the relative Okinawan mortality advantage in older cohorts progressively diminished, approached parity among cohorts born around 1940, and reversed among later-born cohorts (Figure 5).

This pattern independently reproduces the generational divide reported by Poulain and Herm [1] and aligns it with population-level nutrition-transition timing. Using approximately 1970 as a marker of Okinawa’s transition, cohorts near the mortality crossover would have been approximately 25–30 years old at transition, whereas earlier cohorts with a larger mortality advantage had experienced a longer pre-transition period. This timing is descriptive and does not identify an optimal or harmful transition age.

The cohort gradient is consistent with the proposed Lean-to-Rich versus Rich-to-Rich contrast: cohorts with a substantial preceding Lean period retained the most favorable relative mortality, whereas cohorts exposed to the post-transition environment from younger ages lost that advantage. Because the same historical structure also lengthens cumulative Rich exposure in later-born cohorts, however, the gradient does not by itself distinguish a sequence effect from cumulative exposure (Section 6.1).

### 5.2. Contemporary Age-Specific Pattern

The 2020 Prefectural Life Tables provide a complementary cross-sectional observation [20]. Okinawa retained a more favorable relative position at older than at younger ages: among men, remaining life expectancy at age 75 was 12.93 years in Okinawa versus 12.47 years nationally, whereas at age 40, it was 41.71 versus 42.43 years; women showed the same direction. A cross-sectional life table cannot separate age, period, and cohort effects [21]; these age gradients are therefore considered observations consistent with, rather than evidence proving, the proposed birth-cohort × nutrition-transition framework, although they are concordant with the synthetic-cohort findings. Formal birth-cohort analyses provide stronger supporting evidence but remain observational [1].

The two additional analyses are directionally concordant. In the 1975–1979 male decomposition, ages 20–39 already contributed negatively to the Okinawa–Japan life-expectancy difference, while older ages contributed positively (Figure 4); these younger adults broadly overlap the later-born generations in whom the synthetic-cohort analysis shows attenuation and subsequent reversal of the Okinawan mortality advantage (Figure 5).

### 5.3. Why Prolonged Rich Exposure Could Be Unfavorable

The Rich-to-Rich trajectory potentially carries greater cumulative metabolic burden. Longer exposure to abundant energy availability may increase the lifetime probability of obesity, insulin resistance, diabetes, hypertension, dyslipidemia, cardiovascular disease, and premature mortality [14,17].

Younger postwar Okinawan generations developed substantially greater levels of obesity and cardiometabolic risk than the older generations traditionally associated with exceptional longevity [8,22]. Mortality among younger and middle-aged Okinawan men also became less favorable relative to national values [23].

This pattern is consistent with the possibility that a Rich environment may be beneficial when it follows a Lean phase and provides nutritional reserve, but less favorable when exposure begins earlier and persists for decades. The evidence relevant to both propositions is summarized in Table 1.

## 6. Sequence or Cumulative Exposure? A Critical Alternative Explanation

### 6.1. Sequence Versus Cumulative Exposure

The cohort patterns described above do not uniquely establish an effect of nutritional sequence. A simpler alternative explanation is that they reflect the cumulative duration of exposure to a Rich nutritional environment. Because Okinawa underwent nutritional transition earlier, younger Okinawan cohorts necessarily accumulated longer exposure to the post-transition environment than comparable mainland cohorts [8].

Thus, two related but distinct explanations are compatible with the available observations. Under the sequence hypothesis, a preceding Lean period followed by transition to Rich confers an advantage beyond that attributable to the duration of Rich exposure itself. Under the cumulative-exposure hypothesis, the preceding Lean period has no independent effect; the observed pattern primarily reflects the metabolic consequences of longer exposure to nutritional abundance. The distinction between accumulation, timing, and life-course exposure patterns is well established in life-course epidemiology [24,25].

These mechanisms are intrinsically correlated in the Okinawan setting. Because each region underwent a single population-level transition, earlier transition simultaneously shortens the preceding Lean period and lengthens subsequent Rich exposure, and both are structurally linked to birth cohort and attained age. The aggregate data contain no comparison in which cumulative Rich exposure is similar but the preceding Lean period differs, and they therefore cannot separate the two models. The cohort gradient is consistent with a life-stage-dependent effect of nutritional transition, but it is equally compatible with a cumulative-exposure model in which a pre-existing survival advantage of older Okinawan cohorts was progressively eroded. These cohorts were already advantaged in 1975–1979 (Section 4.2), and the origin of that advantage cannot be resolved here, because the prewar evidence is heterogeneous [1,6] and the oldest cohorts were also shaped by wartime and postwar survival selection. Importantly, a pronounced cohort gradient was also evident among women, indicating that the pattern is not confined to the stronger male deterioration. This non-identifiability is a central limitation of the present framework rather than a conventional residual-confounding issue.

A critical test will therefore be whether a preceding Lean period predicts healthy longevity after the duration and intensity of subsequent Rich exposure are accounted for; study designs capable of this are outlined in Section 9.

### 6.2. Relation to DOHaD and the Developmental Mismatch Hypothesis

At first sight, the Lean-to-Rich hypothesis may appear to conflict with the developmental origins of health and disease (DOHaD) framework and the related thrifty-phenotype hypothesis. These established concepts propose that nutritional constraint during fetal or early postnatal development followed by greater nutritional abundance may create a developmental mismatch that increases later cardiometabolic risk [26,27,28].

The Lean-to-Rich hypothesis should therefore not be interpreted as proposing that fetal, infant, or early-childhood nutritional deprivation followed by nutritional abundance is beneficial. The historical Okinawan observations underlying the present hypothesis concern a substantially broader and apparently later life-course exposure window. The older cohorts that subsequently exhibited the strongest longevity advantage had spent childhood, adolescence, and a substantial portion of adulthood before the major postwar nutritional transition, whereas later-born cohorts encountered the changing postwar food environment at progressively younger ages [1,8].

This generational pattern provides no evidence that an early developmental Lean-to-Rich transition is beneficial. If anything, the loss of Okinawa’s mortality advantage among progressively later-born cohorts is compatible with the possibility that transition during earlier developmental periods may have different, and potentially adverse, consequences [1].

Thus, the apparent contrast between Lean-to-Rich and DOHaD may reflect differences in exposure window rather than opposing biological principles. DOHaD primarily concerns nutritional programming during developmental critical periods and subsequent disease risk [27,28], whereas the present hypothesis asks whether a later transition between nutritional environments may influence overall healthy longevity through a balance between cumulative metabolic burden and later nutritional or physiological reserve.

The Okinawan historical evidence does not identify an optimal age for such a transition. It does, however, caution against extrapolating the proposed Lean-to-Rich advantage to fetal or early-childhood undernutrition. Determining whether there is an age or developmental boundary below which Lean-to-Rich becomes unfavorable rather than favorable represents an important and testable extension of the hypothesis.

## 7. A Unified Interpretation of Okinawa’s Rise and Loss of Longevity Advantage

### 7.1. Phase 1: Emergence of the Okinawan Advantage

Okinawa underwent its nutrition transition earlier than mainland Japan [8]. Older Okinawan cohorts therefore approximated a Lean-to-Rich trajectory, whereas comparable mainland cohorts remained in a relatively Lean environment for longer.

If Lean-to-Rich is more favorable than persistent Lean-to-Lean, the earlier emergence of Lean-to-Rich cohorts in Okinawa could have contributed to a temporary relative longevity advantage. The relatively rapid widening of the period life-expectancy gap after the transition should not be interpreted as evidence that nutritional change produced a mortality benefit within only a few years. The age decomposition shows that the widening from 1975–1979 to 1985–1989 was concentrated largely at older ages; among women, the contribution from ages ≥85 increased from approximately +0.53 to +0.96 years (Figure 4). This is compatible with already advantaged older, pre-transition cohorts progressively entering the oldest age groups, rather than requiring an immediate effect of dietary change.

### 7.2. Phase 2: Mainland Japan Catches Up

Mainland Japan subsequently underwent its own nutrition transition [8]. Cohorts that had previously remained in a relatively Lean nutritional environment increasingly experienced nutritional enrichment, thereby generating Lean-to-Rich trajectories with a temporal lag relative to Okinawa.

If this trajectory is favorable, the later emergence of Lean-to-Rich cohorts in mainland Japan could have contributed to national improvements in mortality and, consequently, to narrowing of the Okinawan advantage. This is consistent with Okinawa’s absolute life expectancy continuing to increase while its relative advantage declined [7].

### 7.3. Phase 3: Rich-to-Rich Cohorts Emerge in Okinawa

Meanwhile, successively younger Okinawan birth cohorts experienced progressively less of the earlier Lean nutritional environment and increasingly prolonged exposure to the post-transition environment [8]. Their life-course nutritional trajectories therefore increasingly approximated Rich-to-Rich rather than Lean-to-Rich.

The convergence may therefore reflect three concurrent processes: a declining contribution of Lean-to-Rich cohorts in Okinawa, an increasing contribution of Lean-to-Rich cohorts in mainland Japan, and the emergence of increasingly Rich-to-Rich cohorts in Okinawa. Together, these processes could contribute to the observed convergence in longevity.

This unified framework differs from an interpretation in which the loss of Okinawa’s longevity advantage is attributed simply to “westernization.” Westernization may indeed have contributed to unfavorable health trends in younger Okinawan generations [1,8], but the present hypothesis adds a temporal and life-course dimension: the same broad nutritional enrichment may have different consequences depending on the nutritional environment that preceded it and the stage of life at which it occurred.

The rise and subsequent loss of Okinawa’s relative longevity advantage may therefore represent two phases of the same historical process rather than two unrelated phenomena. This life-course formulation is the principal theoretical contribution of the Lean-to-Rich Healthy Aging Hypothesis.

## 8. Alternative Explanations and Limitations

Several limitations are addressed where they arise in the preceding sections: the contribution of early-life mortality to life expectancy at birth (Section 4.2), the heterogeneity of prewar life-table evidence (Section 1), the limits of BMI as a marker of nutritional status (Section 4.3), the cross-sectional nature of contemporary age gradients (Section 5.2), the inability of ecological data to separate sequence from cumulative exposure (Section 6.1), the unspecified timing of transition (Section 3), and the developmental boundary (Section 6.2). Further limitations are as follows.

First, the evidence is primarily historical and ecological. Individual nutritional trajectories were not measured; cohorts were assigned to approximate trajectories on the basis of the nutritional environments prevailing in different historical periods, and considerable heterogeneity existed within each cohort. Population-level associations therefore cannot be assumed to apply to individuals [24,29].

Second, Okinawa’s postwar nutrition transition coincided with profound changes in medical care, sanitation, infectious disease control, education, income, housing, transportation, occupation, physical activity, smoking, alcohol consumption, and social structure, and U.S. administration from 1945 to 1972 created distinctive socioeconomic and health-care conditions [1,8]. These factors could independently generate or modify the observed mortality patterns. The regional time lag is therefore a hypothesis-generating historical circumstance, not a controlled natural experiment or quasi-randomized exposure, and the temporal correspondence cannot establish that nutrition was the principal causal determinant, or that nutritional sequence provides a better explanation than these competing factors.

Third, the fat-energy ratio captures only one dimension of a multidimensional transition in which total energy and protein intake, carbohydrate quality, micronutrient adequacy, food processing, dietary diversity, and physical activity changed concurrently [8].

Fourth, the different male and female trajectories suggest that nutrition alone is unlikely to account for the entire pattern. Smoking and alcohol exposure, physical activity and automobilization, occupational and socioeconomic change, health-care access and utilization, low birth weight, migration, and sex-specific cardiometabolic risk may all contribute [1,8,23]. The stronger deterioration among men is compatible with important non-nutritional modifiers; however, the birth-cohort gradient is also clearly present among women (Figure 5). The Lean-to-Rich framework is therefore proposed as one life-course component of a multifactorial historical process, not as a complete explanation of Okinawa’s mortality history.

Fifth, historical claims of exceptional survival to extreme ages may be sensitive to age validation and the completeness of vital registration [30]. Such concerns are particularly relevant to studies based on counts of centenarians or exceptionally old individuals. However, the principal quantitative observations used in the present framework derive from official prefectural life tables and age-specific mortality-rate data rather than from centenarian or supercentenarian counts [7]. Concerns regarding extreme-age validation therefore do not directly invalidate the analyses presented here, although they warrant caution when the broader Okinawan longevity literature is interpreted.

Finally, Lean-to-Lean, Lean-to-Rich, and Rich-to-Rich are conceptual archetypes rather than discrete biological categories. Actual nutritional histories are continuous and multidimensional; an individual may be relatively Lean in energy but Rich in protein or micronutrients. Any potentially favorable transition is likely to depend on age, sex, baseline nutritional status, physical activity, body composition, and the foods or nutrients involved; the historical data do not define a universal schedule. Future studies should therefore use repeated measures of diet, body composition, metabolic status, and functional reserve rather than assigning categories by historical period, location, or BMI.

Taken together, these limitations preclude causal inference from the current evidence, but they also define the conditions under which the hypothesis can be tested and potentially falsified. The present framework should therefore be regarded not as evidence that Lean-to-Rich is an established optimal nutritional trajectory, but as a hypothesis generated from Okinawa’s unusual historical experience that warrants evaluation using individual-level life-course data.

## 9. Testing the Lean-to-Rich Hypothesis

The Lean-to-Rich Healthy Aging Hypothesis generates several testable predictions and should be evaluated against alternative life-course models rather than solely by seeking observations consistent with it.

First, longitudinal studies should reconstruct individual nutritional trajectories from repeated information on diet, body composition, physical activity, metabolic status, and socioeconomic conditions, allowing Lean-to-Lean, Lean-to-Rich, and Rich-to-Rich trajectories to be modeled directly [24,25]. The central prediction is that a substantial Lean period followed by nutritional enrichment will be associated with more favorable healthy-aging outcomes than either persistent Lean or prolonged Rich exposure, after major confounders are addressed.

Second, analyses should explicitly compare sequence models with accumulation models [24,25]. The sequence hypothesis would be weakened if the preceding Lean period showed no independent association with healthy longevity after cumulative Rich exposure and relevant confounders were controlled; if outcomes were explained entirely by the duration or intensity of Rich exposure, an accumulation model would be favored.

Third, direct comparison of the proposed trajectories provides another falsification test. The hypothesis would be challenged if Rich-to-Rich trajectories consistently produced healthy-aging outcomes comparable to or more favorable than Lean-to-Rich trajectories across independent populations. Similarly, if persistent Lean-to-Lean trajectories were consistently associated with outcomes equal to or better than Lean-to-Rich after accounting for illness-related weight loss and other sources of reverse causation, the proposed advantage of transition would not be supported.

Fourth, designs that break the structural coupling between the preceding Lean period and subsequent Rich exposure are particularly informative. Individuals from the same source population who migrate to nutritionally richer environments at different ages undergo transition at different life stages, and migrant studies have long been used to examine how environmental change modifies chronic-disease risk [31,32]. Migrant, sibling, or family-based comparisons of individuals with similar cumulative Rich exposure but different durations of preceding Lean exposure could distinguish a sequence effect from cumulative exposure.

Fifth, populations in East Asia and other regions that underwent rapid economic and nutritional development at different historical times provide an opportunity to test the transition-timing prediction generated from Okinawa. Nutrition transition has occurred at markedly different rates and historical periods across populations [33,34]. If the Lean-to-Rich hypothesis has broader validity, regions with earlier nutritional transitions should initially generate Lean-to-Rich cohorts sooner, followed later by increasing Rich-to-Rich exposure among younger cohorts, whereas regions undergoing later transitions should show corresponding cohort patterns with a temporal lag. Failure to observe such temporal displacement across populations with clearly different transition timings would weaken the proposed birth-cohort × transition-timing framework.

Sixth, the hypothesis should be tested against outcomes beyond total mortality, including disability-free survival, frailty, sarcopenia, cardiovascular disease, diabetes, cognitive impairment, and functional independence. Healthy aging is increasingly conceptualized in terms of maintaining functional ability rather than survival alone [35]. A nutritional trajectory that improves survival while substantially worsening disability or chronic disease would not necessarily support a Healthy Aging hypothesis. The appropriate endpoint should therefore ultimately incorporate both longevity and preserved function.

Taken together, the hypothesis would be most strongly supported if a preceding Lean nutritional environment predicted favorable healthy-aging outcomes independently of cumulative Rich exposure, if Lean-to-Rich trajectories outperformed both Lean-to-Lean and Rich-to-Rich trajectories, and if the predicted cohort patterns shifted temporally across populations according to the historical timing of nutritional transition. Conversely, failure of these predictions—particularly the absence of an independent sequence effect after cumulative exposure is accounted for—would favor simpler alternative explanations and would require modification or rejection of the Lean-to-Rich sequence hypothesis.

## 10. Lessons from Okinawa’s History for Future Healthy Aging

Much of nutrition research asks whether restriction or abundance is preferable, whether a particular BMI is optimal, or whether one dietary pattern is associated with longer life [13,14,18]. The Lean-to-Rich hypothesis instead asks whether the nutritional environment that is favorable at one life stage may differ from that which is favorable later. Its central proposition is neither that Lean is better than Rich nor the reverse, but that a transition from Lean to Rich may be more favorable than remaining in either state throughout the life course. This may help reconcile apparently conflicting observations: lower cumulative metabolic exposure may reduce cardiometabolic risk [13,14], whereas adequate nutritional and physiological reserve may become increasingly important for resilience, muscle preservation, recovery from illness, and survival at older ages [16]. Whether Lean-to-Rich actually combines these properties remains unknown.

Okinawa is used here as a hypothesis-generating natural setting, not as a dietary template. Its specific diet, transition timing, and historical circumstances are not assumed to be transferable, and the hypothesis implies no recommendation to manipulate nutrition at a particular age. Okinawa is informative because its postwar history produced a measurable temporal separation in nutritional transition from mainland Japan [8], which may have made life-course differences visible at the population level.

## 11. Conclusions

Okinawa’s exceptional longevity cannot be understood fully by assuming that its traditional Lean nutritional environment was uniformly beneficial or that subsequent nutritional enrichment was uniformly harmful. Okinawa underwent postwar nutritional enrichment earlier than mainland Japan [8], creating a temporal separation in life-course nutritional trajectories across successive birth cohorts. During the subsequent decades, Okinawa developed a marked relative longevity advantage, which later diminished as longevity elsewhere in Japan continued to improve and younger Okinawan generations experienced the post-transition nutritional environment from progressively earlier stages of life [1,7].

These historical observations suggest a unified interpretation. The earlier nutrition transition in Okinawa may have generated Lean-to-Rich cohorts before comparable trajectories became common in mainland Japan, potentially contributing to the emergence of Okinawa’s relative longevity advantage. The later emergence of Lean-to-Rich trajectories in mainland Japan may have contributed to national catch-up, while progressively younger Okinawan cohorts increasingly approximated Rich-to-Rich trajectories. Together, these processes may have contributed to the subsequent convergence in longevity between Okinawa and Japan as a whole.

We therefore propose the Lean-to-Rich Healthy Aging Hypothesis: a life-course trajectory beginning in a relatively Lean nutritional environment and subsequently transitioning to a Richer environment may be more favorable for healthy longevity than either persistent relative nutritional restraint or prolonged nutritional abundance.

The hypothesis does not define the optimal timing, magnitude, duration, or composition of the transition, should not be extrapolated to fetal or early-childhood deprivation followed by abundance [26,27,28], and cannot yet be distinguished from cumulative Rich exposure using ecological data. These alternatives require testing with individual-level life-course data and natural experiments in which transition timing and exposure duration can be separated.

Rather than establishing causality, the proposed framework transforms Okinawa’s unusual historical experience into a series of testable predictions. Its principal contribution is to shift attention from whether a Lean or Rich nutritional environment is inherently favorable to whether the sequence and duration of nutritional environments across the life course influence healthy longevity. Although generated from Okinawa’s unique postwar history, the Lean-to-Rich Healthy Aging Hypothesis may provide a broader framework for understanding healthy aging in populations worldwide that have undergone nutritional transitions at different times and rates.

## Figures and Tables

**Figure 1:**
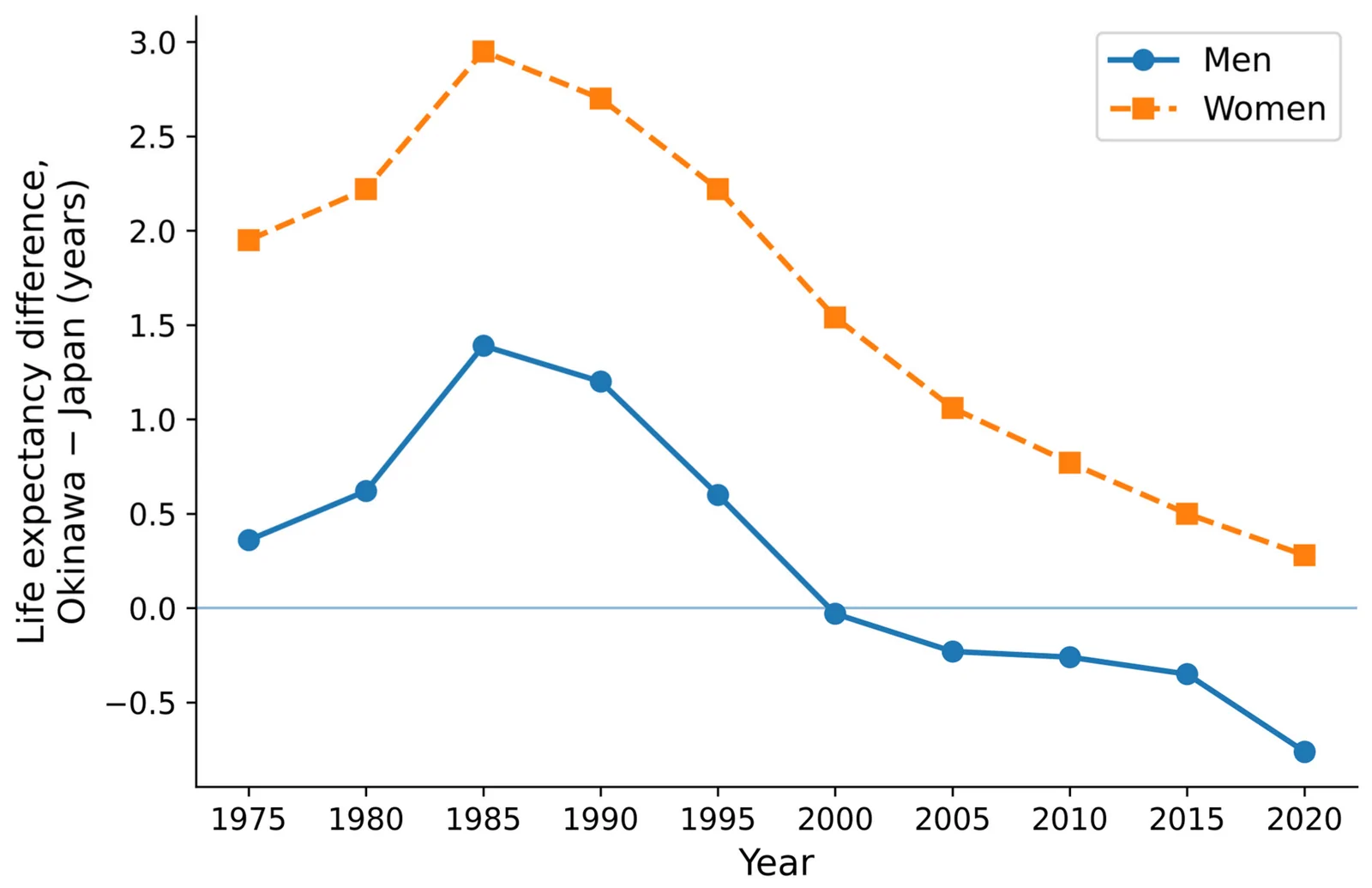
Historical change in Okinawa’s longevity advantage relative to Japan, 1975–2020. Longevity advantage was calculated as life expectancy at birth in Okinawa minus the corresponding national life expectancy for each sex and year. Positive values indicate longer life expectancy in Okinawa than the national average, whereas negative values indicate shorter life expectancy. Data were obtained from the Ministry of Health, Labour and Welfare, Japan, *Trends in Life Expectancy by Prefecture*, Table 80000 [7], and differences were calculated by the author. No statistical modeling or smoothing was applied.

**Figure 2:**
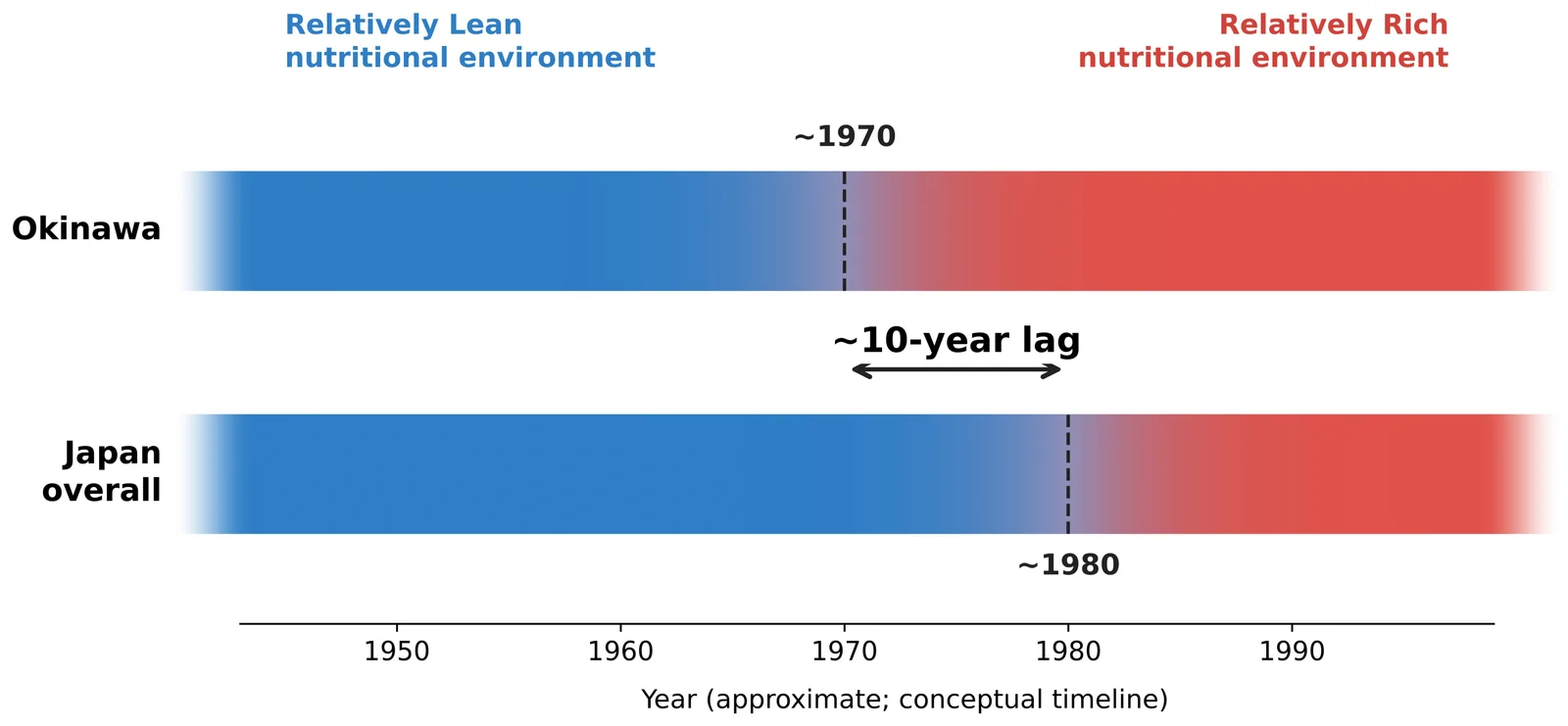
Conceptual schematic of the earlier postwar nutrition transition in Okinawa than in Japan overall. The midpoint of each gradient is placed at the approximate regional transition year, based on Todoriki et al. [8], who reported that the fat-energy ratio reached approximately 25% around 1970 in Okinawa and around 1980 in Japan overall. The gradients represent gradual population-level change rather than precisely dated boundaries; the faded ends indicate that the relatively Lean environment continued before, and the relatively Rich environment after, the period shown. The blue-to-red gradient and the terms “Lean” and “Rich” are conceptual representations introduced in the present hypothesis and are not quantitative data or terminology derived from Todoriki et al.

**Figure 3:**
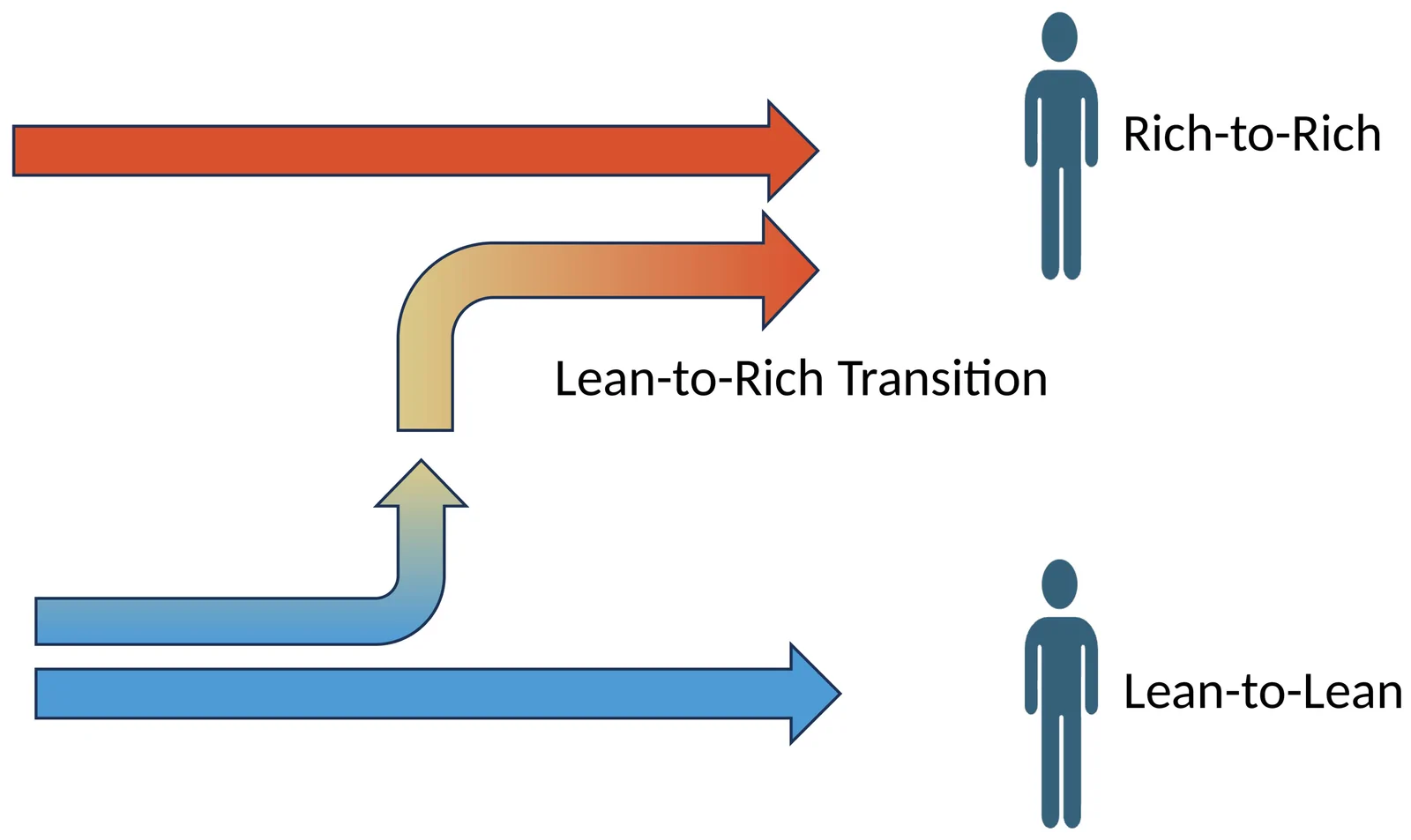
Three conceptual life-course nutritional trajectories: Lean-to-Lean, Lean-to-Rich, and Rich-to-Rich.

**Figure 4:**
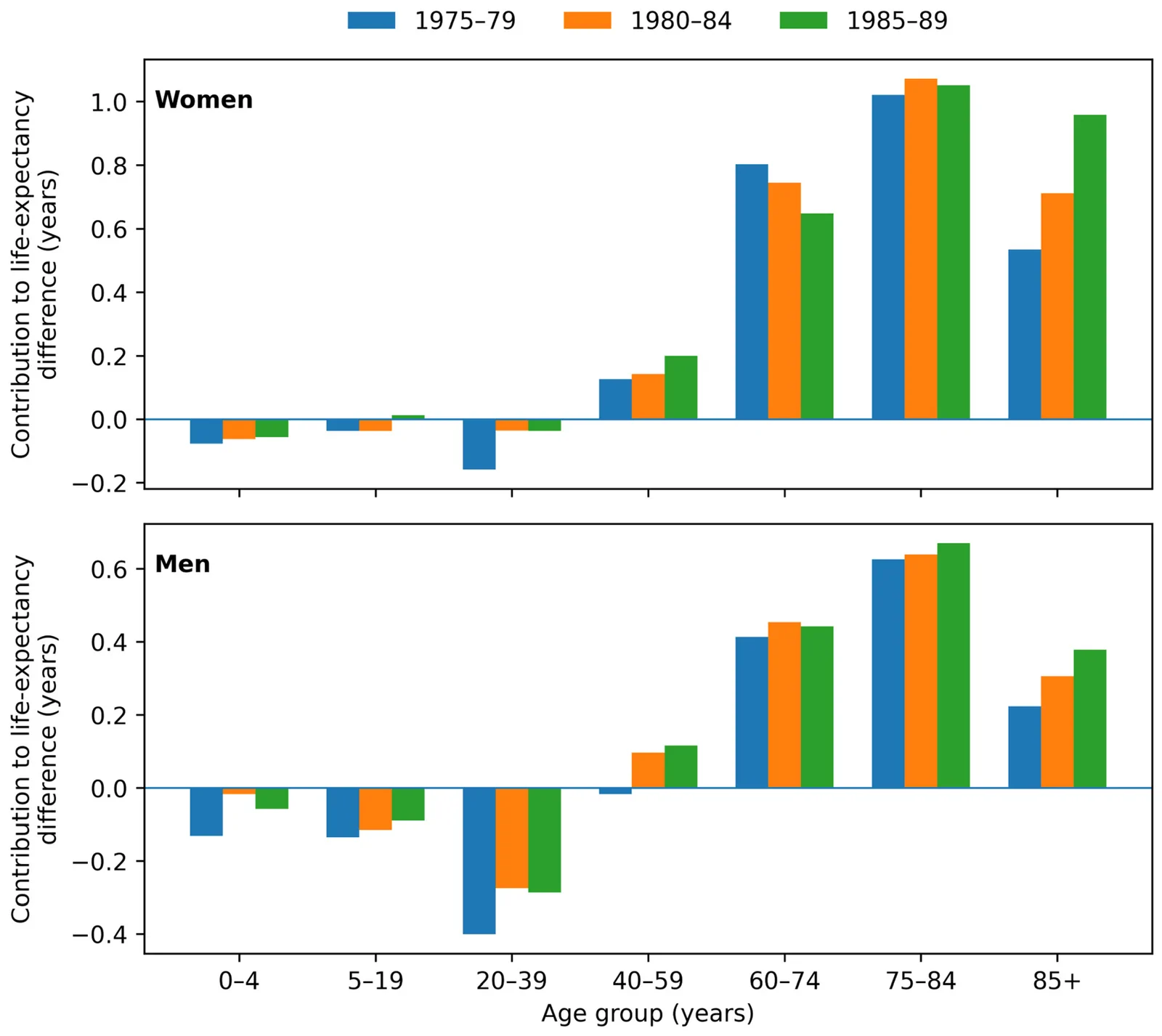
Age-specific contributions to the Okinawa–Japan difference in life expectancy. Contributions were estimated from Japanese Mortality Database 5-year life tables by symmetric stepwise replacement (Data and Methods for the Mortality Reanalyses Section). Positive values indicate contributions favoring longer life expectancy in Okinawa; negative values indicate contributions offsetting the Okinawan advantage.

**Figure 5:**
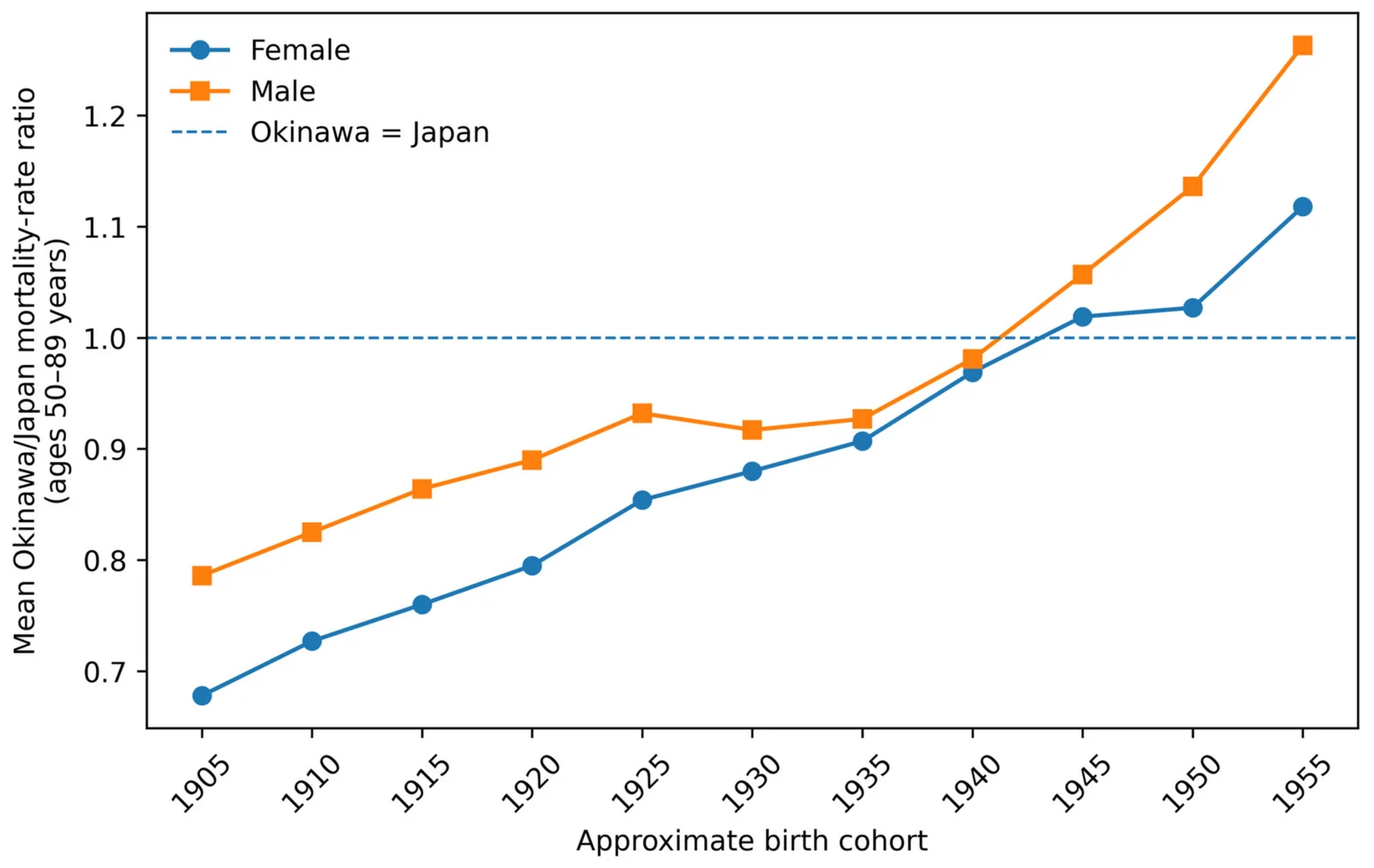
Relative mortality across synthetic birth cohorts in Okinawa versus Japan. Sex-specific mean Okinawa-to-Japan all-cause mortality-rate ratios at ages 50–89 years, reconstructed from Japanese Mortality Database 5-year period × 5-year age-group data (Data and Methods for the Mortality Reanalyses Section). Values below 1.0 indicate lower mortality in Okinawa. Synthetic cohorts are population-level reconstructions rather than individual longitudinal follow-up.

**Table 1:** Evidence relevant to the Lean-to-Rich Healthy Aging Hypothesis, organized by evidentiary role. Direct evidence refers to the historical demographic pattern and nutrition-transition timing, not to causal superiority of Lean-to-Rich. No study directly compares all three proposed trajectories.

Evidence Level	Evidence/Source	Main Observation	Interpretation for Lean-to-Rich
Direct historical pattern	MHLW life tables; JMD mortality reanalysis [7,9]	Relative Okinawan longevity rose and later converged; synthetic cohorts show progressive loss and reversal of mortality advantage in later-born cohorts.	Establishes the demographic pattern to be explained; does not establish nutritional causality.
Direct age-specific pattern	JMD life-table decomposition [9,10,11,12]	During 1975–1989, the Okinawa–Japan life-expectancy advantage was concentrated predominantly at ages ≥60 years; ages 0–4 contributed slightly negatively.	Shows that the historical advantage reflected older-age survival rather than lower infant/child mortality; does not identify its cause.
Direct transition timing	Todoriki et al. [8]	Postwar dietary transition occurred earlier in Okinawa than in Japan overall.	Establishes the regional timing contrast used to infer differing life-course exposure histories.
Indirect support	Older-adult nutrition/BMI literature [15,16,18]	Low body mass and inadequate nutritional reserve can be unfavorable in older adults.	Supports plausibility that persistent Lean status need not remain optimal throughout life.
Indirect support	Obesity/metabolic-burden literature [14,17,22]	Prolonged adiposity and metabolic burden are associated with adverse outcomes; younger Okinawan cohorts developed greater metabolic burden.	Supports plausibility that prolonged Rich exposure may be unfavorable.
Consistent, non-specific	Poulain & Herm [1] and Okinawan historical observations	Older cohorts show favorable mortality, whereas later cohorts show progressively less favorable mortality.	Consistent with a cohort × transition-timing framework but also compatible with cumulative exposure and other factors.
Hypothesis generated here	Lean-to-Rich Healthy Aging Hypothesis	Integrates transition timing, older-age survival, cohort mortality, and life-course trajectory.	Predicts, but does not yet prove, an advantage of Lean-to-Rich over persistent Lean-to-Lean or prolonged Rich-to-Rich.

## Data Availability

No new primary data were collected. The additional analyses use publicly available Japanese Mortality Database data for Okinawa Prefecture and Japan overall (JMD version 005_002), including sex-specific 5-year period death-rate tables and life tables. The synthetic birth-cohort ratios and age-specific life-expectancy decomposition can be reproduced using the procedures described in the Data and Methods for the Mortality Reanalyses Section.
